# Multicenter study of the natural history and therapeutic responses of patients with chikungunya, focusing on acute and chronic musculoskeletal manifestations – a study protocol from the clinical and applied research in Chikungunya (REPLICK network)

**DOI:** 10.1186/s12879-023-08292-y

**Published:** 2023-07-28

**Authors:** Giselle da Silva Duarte, Alexandra D. Jones, Luciano Pamplona de Goes Cavalcanti, Moacyr Jesus Barreto de Melo Rêgo, Guilherme S. Ribeiro, Rosemary J. Boyton, Dhelio Batista Pereira, Julio Henrique Rosa Croda, Fabio Trindade Maranhão Costa, Angela Pinto Duarte, Marcia Edilaine Lopes Consolaro, Rodrigo Guerino Stabeli, Fábio Juliano Negrão, Jose Luiz Proenca-Modena, Juan Miguel Villalobos-Salcedo, Geraldo da Rocha Castelar Pinheiro, Amanda Pinheiro de Barros Albuquerque, Francisca Kalline de Almeida Barreto, Jose Moreira, Idalina Cristina Ferrari, Patricia Martinez Évora, Vânia Ramos Sela da Silva, Marcus Vinicius Guimarães Lacerda, Daniel M. Altmann, REPLICK network, Thyago Henrique Pereira dos Santos, Fernanda Montenegro de Carvalho Araujo, Luis Arthur Brazil Gadelha Farias, Lara Moreira Teles de Vasconcelos, Brena Ferreira dos Santos, Shamyr Sulyvan de Castro, Marina Carvalho Arruda Barreto, Ileana Pitombeira Gomes, Jobson Lopes de Oliveira, Thayanne Vitoria Nunes Pinheiro, Samara Bezerra Guedes das Neves, Carla Cristiana Judice, Gabriela Fabiano de Souza, Matheus Cavalheiro Martini, Deusilene Souza Vieira Dall’Acqua, Marcela Macedo de Oliveira, Eduardo Garbin, Alexia Martines, Maira Galdino da Rocha Pitta, Luiz Demarchi, Caroline Santos Soares, Patricia Brasil, Karen Trinta, Claudia Pereira, André M. Siqueira

**Affiliations:** 1 Laboratório de Pesquisa Clínica Em Doenças Febris Agudas, Instituto Nacional de Infectologia Evandro Chagas, Fundação Oswaldo Cruz, Rio de Janeiro, Brazil; 2 University of California, Berkeley, USA; 3 Universidade Federal Do Ceará, Fortaleza, Brazil; 4 Departamento de Bioquímica, Universidade Federal de Pernambuco, Recife, Brazil; 5 Instituto Gonçalo Moniz, Fundação Oswaldo Cruz, Salvador, Brazil; 6 Imperial College of London, London, UK; 7 Centro de Pesquisa Em Medicina Tropical de Rondônia, Porto Velho, Brazil; 8 Fundação Oswaldo Cruz, Mato Grosso Do Sul, Campo Grande, Brazil; 9 Universidade Estadual de Campinas, Campinas, Brazil; 10 Hospital das Clínicas, Universidade Federal de Pernambuco, Recife, Brazil; 11 Departamento de Análises Clínicas E Biomedicina, Universidade Estadual de Maringá, Maringá, Brazil; 12 Plataforma de Medicina Translacional, Fundação Oswaldo Cruz, São Paulo, Brazil; 13 Universidade Federal da Grande Dourados, Dourados, Brazil; 14 Fundação Oswaldo Cruz, São Paulo, Brazil; 15 Universidade Estadual Do Rio de Janeiro, Rio de Janeiro, Brazil; 16 Fundação de Medicina Tropical Dr Heitor Vieira Dourado, Manaus, Brazil

**Keywords:** Chikungunya, Cohort, Brazil, Rheumatic manifestations

## Abstract

**Background:**

Chikungunya is associated with high morbidity and the natural history of symptomatic infection has been divided into three phases (acute, post-acute, and chronic) according to the duration of musculoskeletal symptoms. Although this classification has been designed to help guide therapeutic decisions, it does not encompass the complexity of the clinical expression of the disease and does not assist in the evaluation of the prognosis of severity nor chronic disease. Thus, the current challenge is to identify and diagnose musculoskeletal disorders and to provide the optimal treatment in order to prevent perpetuation or progression to a potentially destructive disease course.

**Methods:**

The study is the first product of the Clinical and Applied Research Network in Chikungunya (REPLICK). This is a prospective, outpatient department-based, multicenter cohort study in Brazil. Four work packages were defined: i. Clinical research; ii) Translational Science – comprising immunology and virology streams; iii) Epidemiology and Economics; iv) Therapeutic Response and clinical trials design. Scheduled appointments on days 21 (D21) ± 7 after enrollment, D90 ± 15, D120 ± 30, D180 ± 30; D360 ± 30; D720 ± 60, and D1080 ± 60 days. On these visits a panel of blood tests are collected in addition to the clinical report forms to obtain data on socio-demographic, medical history, physical examination and questionnaires devoted to the evaluation of musculoskeletal manifestations and overall health are performed. Participants are asked to consent for their specimens to be maintained in a biobank. Aliquots of blood, serum, saliva, PAXgene, and when clinically indicated to be examined, synovial fluid, are stored at -80° C. The study protocol was submitted and approved to the National IRB and local IRB at each study site.

**Discussion:**

Standardized and harmonized patient cohorts are needed to provide better estimates of chronic arthralgia development, the clinical spectra of acute and chronic disease and investigation of associated risk factors. This study is the largest evaluation of the long-term sequelae of individuals infected with CHIKV in the Brazilian population focusing on musculoskeletal manifestations, mental health, quality of life, and chronic pain. This information will both define disease burden and costs associated with CHIKV infection, and better inform therapeutic guidelines.

**Supplementary Information:**

The online version contains supplementary material available at 10.1186/s12879-023-08292-y.

## Background

Chikungunya disease is caused by the chikungunya virus (CHIKV), a mosquito-borne alphavirus belonging to the family *Togaviridae*. Current estimation projects that 1.3 billion people are at risk of CHIKV infection [1] as the mosquito vectors (*Aedes aegypti and Ae albopictus*). It is estimated to have a high attack rate ranging from 5.3 to 40% and asymptomatic infection ranges from 35–82% [2]. Four major genotypes of CHIKV are now recognized – the Asian, the West African, the East-Central South African genotypes, and a new lineage – the Indian Ocean, which emerged during the 2006 Reunion Island outbreak. The disease is associated with high morbidity due to the musculoskeletal commitment that can be delibitating to individuals apart from a raised awareness of the associated mortality, based specially on evidence of increased excess mortality during outbreaks [3, 4].

The first case of autochthonous transmission of the CHIKV in Brazil was reported in September 2014, in Oiapoque, in the northern state Amapa [5]. In the same month, an outbreak was also reported in Feira de Santana, in the northeastern state Bahia [6]. Phylogenetically studies suggest two CHIKV lineages were introduced in Brazil – Asian and ECSA [7]. The large (immune-naive to CHIKV) population density, an abundance of urban conglomerates, and optimal climate conditions for proliferation of *A aegypti* in the Brazilian territory favored the emergence of a massive CHIKV outbreaks in distinct areas of Brazil and an even more worrying possibility of its expansion to several other área [8] During the highest Brazilian outbreak, in 2016, 277.882 probable CHIKV cases were reported, corresponding to an incidence rate of 134.8 cases/100.000 population [9]. Lately, in 2021, a new wave of CHIKV cases were observed, counting with 96.288 cases (an incidence rate of 45.1 cases/100.000 population) [9].

Patients infected with CHIKV typically present with a rapid-onset febrile illness, characterized by intense asthenia, arthralgia, myalgia, headache, and rash. Soon after the onset of fever, severe myalgias and arthralgias may occur; frequently of an intensity that can result in severe impairment of the individuals mobility and impacting his/her autonomy to perform work activities and even self-care. Joint pain is usually symmetric, affecting primarily peripheral joints and to a lesser extent, involvement of the axial skeleton [10]. Periarticular edema, tenosynovitis and acute arthritis may also occur, particularly in the interphalangeal joints, wrists, and ankles, as well as pain along with ligament and tendon insertions.

The natural history of symptomatic CHIKV infection has been divided into three phases – acute, post-acute, and chronic—according to the duration of musculoskeletal symptoms. Although this classification has been designed to help guide therapeutic decisions, it does not necessarily encompass the complexity of the clinical expression of the disease and does not assist in the evaluation of the prognosis of severity nor chronic disease. As such, subjects presenting with severe acute forms, rare yet associated with fatal outcomes, are not adequately addressed in the current proposal and the need for improvement has been raised by our group [11].

Although most of the textbooks and guidelines describe chronic chikungunya as an inflammatory arthritis resembling rheumatoid arthritis [12], around 95% of patients who maintain musculoskeletal pain beyond three months after symptoms onset do not develop polyarthritis, and have substantial clinical improvement with prolonged administration of nonsteroidal anti-inflammatory), analgesics, and local treatment, including physiotherapy [13]. In contrast, around 5% of patients meet the criteria for chronic inflammatory rheumatism (i.e., rheumatoid arthritis, spondylarthritis, or undifferentiated polyarthritis), may benefit from antirheumatic drugs (such as methotrexate), as recommended by national guidelines [14, 15]. An understanding of the clinical disease spectrum since symptoms onset and of disease pathogenesis is essential to improve therapeutic strategies for millions of affected patients worldwide. Moreover, there are individuals that present neuropathic pain with clear neural involvement. It is needed therefore to acknowledge that chikungunya presentation can have at least three distinct phenotypes occurring isolated or in combination: i) inflammatory joint disease – with clinical and laboratory signs of arthritis,ii) mechanical arthralgia – joint pain without inflammatory oedema and/or laboratorial inflammation markers,iii) neuropathic pain – with neural involvement and response to neuroleptic therapies.

The proportion of patients with persistent polyarthralgia following the acute phase of chikungunya varies according to studies [16]. The lack of homogeneity on the definitions of outcome, high variability of instruments and methods applied to measure chronic disease and selection and observation bias result in a wide range of estimates. Cohort studies that have systematically followed patients with standardized methods estimate that around 40 to 50% of individuals still are in pain six months after the initial symptoms.

Standardized and harmonized patient cohorts are needed to provide better estimates of chronic arthralgia development, the clinical spectra of acute and chronic disease and investigation of associated risk factors. This information will both define disease burden and costs associated with CHIKV infection, and better inform therapeutic guidelines. Little is known regarding the contribution in pathogenetic mechanisms for chronic chikungunya (including both viral and host factors) responsible for determination of disease progression (i.e. rapid recovery versus development of chronic musculoskeletal symptoms, arthralgia, arthritis, or destructive arthritis). Such information will require both experimental systems and detailed analyses involving appropriate patient cohorts. Other challenges to chikungunya management, given the variety of chronic symptoms associated with CHIKV infection, include the absence of biomarkers to monitor disease progression, the minimal therapeutic options available, and patient’s lack of access to multidisciplinary outpatient clinics.

There are no available evidence-based guidelines with standardized definitions and treatment recommendations for post-CHIKV musculoskeletal disorders. Simple analgesics, NSAIDs and steroids provide relief in most patients, however better-targeted drugs are needed to modify the disability and pain caused by chikungunya providing long-term and sustainable relief and even possibly preventing progression to the chronic phase. Thus, the current challenge for physicians in CHIKV epidemic-prone areas is to identify and diagnose CHIKV musculoskeletal disorders and to provide the optimal treatment in order to prevent perpetuation or progression to a potentially destructive disease course. For this reason, we have built an international network of researchers and clinicians, generating the most substantial ever grouping of researchers targeting understanding of the immune correlates of outcome after CHIKV exposure (International Network for CHIKV investigation has named SPIICA and granted by FAPESP), in progressing towards improved understanding of this disease, leading to earlier and more effective treatment and mechanistic insights into causes of the differential disease outcomes.

Herein, we describe the protocol of this multicenter study which aims to establish a large longitudinal cohort of CHIKV-infected subjects throughout Brazil, aiming to evaluate the proportion of individuals who present with severe forms, evolve to a chronic phase, and the therapeutic response in those with chronic musculoskeletal manifestations. We believe this initiative will have an important role in fulfilling the existing gap in the CHIKV literature by producing evidence-based recommendations and policy for the best understanding of the clinical manifestations associated with CHIKV infection and to assess the applicability of standardized instruments for clinical care and management.

## Methods

### Study hypothesis

We hypothesized that approximately 50% of CHIKV-infected individuals would evolve to chronic disease, and older age and underlying comorbidities would be a decisive factor for progression.

### Study objectives

The study’s primary objective is to investigate the natural history and therapeutic response of musculoskeletal manifestations of individuals infected by CHIKV in the Brazilian territory. Its secondary objectives are: 1) to investigate the risk and prognostic factors associated with the severity and persistence of clinical manifestations, 2) to estimate the financial and psychosocial impact of CHIKV, 3) to examine the pathobiology of CHIKV infection during the acute phase, 4) to identify biomarkers that can establish molecular correlations of disease progression and patterns of persistence of joint pain, and 5) to establish a biobank of well-characterized CHIKV samples (specimens), to support the development of new tools to improve diagnosis and prognosis of CHIKV in endemic regions.

### Study design

This is a prospective, outpatient department-based, multicenter cohort study being conducted at 11 sites in 9 states, encompassing all five macroregions of Brazil, that is, representing distinct epidemiologic scenarios of CHIKV transmission.

**Fig. 1:**
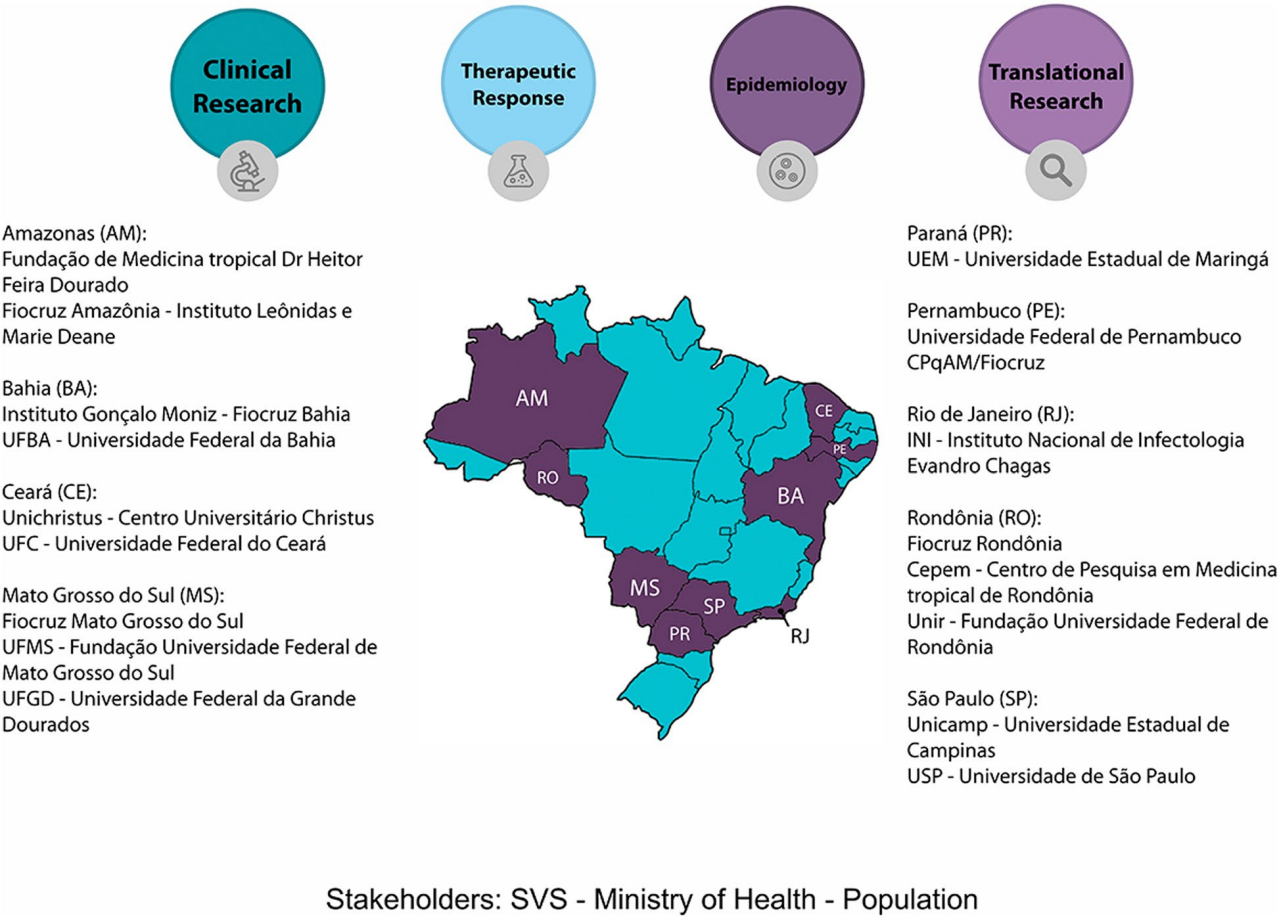
Geographic Distribution of Brazilian Replick sites, work packages and stakeholders

The study is the first product of the Clinical and Applied Research Network in Chikungunya (REPLICK), which was created in 2016 with the objective of promoting integration of expertise and methods to achieve a comprehensive investigation of chikungunya clinical manifestations, therapeutic response and on the mechanisms of pathogenesis to produce evidence that can inform and assist the formulation of public health policies. The network is composed of researchers with expertise in infectious diseases, virology, rheumatology, epidemiology, physiotherapy, pharmacology, and immunology. Four work packages were defined: i. Clinical research; ii) Translational Science – comprising immunology and virology streams; iii) Epidemiology and Economics; iv) Therapeutic Response and clinical trials design. Figure 1

Due to the lack of systematic tools to measure the disease activity of chikungunya, specific groups were formed to decide on the choice of instruments to be used in the protocol (detailed in the study visits section). The study aimed to recruit 2,000 individuals with confirmed CHIKV infection throughout the study sites considering the heterogeneity of transmission and unpredictability of outbreaks occurrence.

### Study participants

The study population consisted of adults (≥ 18 years) who reported recent fever and arthralgia, with suspected arboviral-illness. Exclusion criteria were defined by the following 1) unable to attend the follow-up visits. Eligible participants are identified by attending physicians or research personnel at each participating site. All participants sign informed consent statements before being enrolled in the study activities.

### Definitions

Suspected arboviral illness: individuals presenting to clinical sites with symptoms and signs compatible with arboviral infection, such as fever, arthralgia, myalgia, rash, headache, retro orbital pain, photophobia, back pain, chills, weakness, malaise, nausea, or vomiting.

Confirmed chikungunya infection: individuals with suspected arboviral illness who had

- ● detectable CHIKV RNA in serum or other specimens (i.e., saliva, urine) by RT-PCR, or IgM antibody against CHIKV, or IgM seroconversion between paired samples. CHIKV IgM antibodies were detected by ELISA or rapid tests.

### Study visits

Patients arriving at recruiting centers with suspected arboviral illness are evaluated by study staff and, if eligible, then they are invited to participate in the study throughout ICF signed. Participants receive usual care from their attending physician throughout the study period. Those who accept to be enrolled in the study are interviewed using standardized questionnaires to obtain data on medical history, signs and symptoms, intensity of pain, fatigue, clinical disease activity, activity limitations, heath impact, work/school/housework ability and productivity in addition to medication history. To aid diagnostic workup and determine follow-up strategy, blood samples are collected at enrollment and 10 and 21 days later and tested by molecular and rapid tests. Those with negative results are investigated for other etiologies of febrile illness, and provided care according to the attending physician, and local guidelines. Conversely, those with positive results continue to be followed, being examined by a rheumatologist for careful evaluation of musculoskeletal manifestations, which may be complemented by ultrasound or magnetic resonance imaging.

**Fig. 2:**
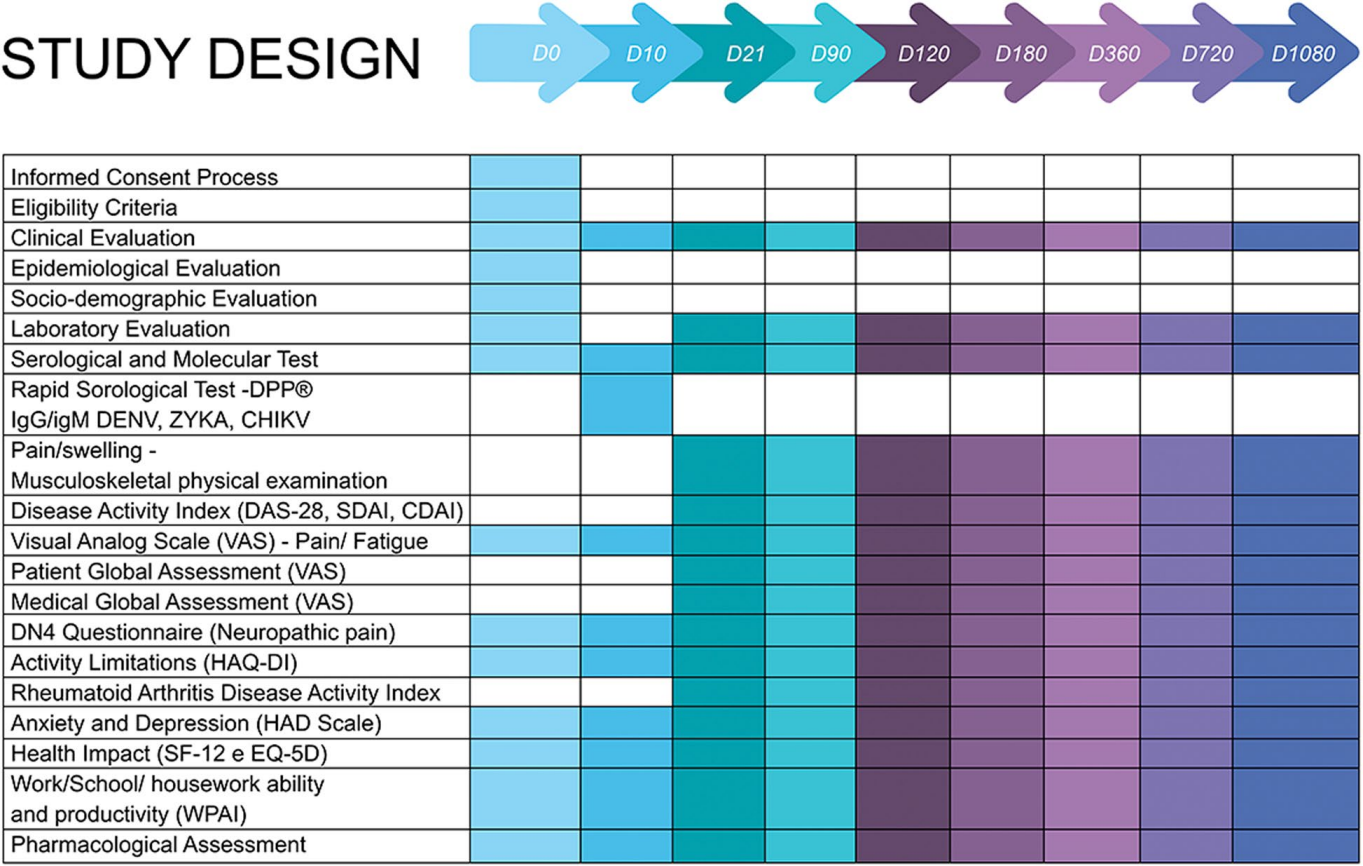
Study Design Chart

Scheduled study appointments with a clinician or rheumatologist following CHIKV infection confirmation, occur on days 21 (D21) ± 7 after enrollment, D90 ± 15, D120 ± 30, D180 ± 30; D360 ± 30; D720 ± 60, and D1080 ± 60 days. On these study visits, questionnaires, clinical reports forms, and a panel of blood tests are collected. Throughout follow-up, participants’ eligibility for the pre-planned sub-studies are assessed, and patients are offered participation in the sub-studies. Figure 2 shows the tasks performed at each study visit.

Unscheduled visits for the study are made under the following conditions: 1) worsening and persistence of pain or articular inflammation, 2) management of an adverse event, 3) worsening of an underlying disease, and 4) at the physician’s discretion if a reevaluation is deemed necessary. The same questionnaires and report forms applied in the scheduled visits are applied in the unscheduled visits. Similarly, blood collection could be required due to worsening or recurrent pain, or joint inflammation. Additional laboratory evaluation may be ordered for patient care and do not represent a protocol deviation.

### Measurement instruments

In addition to the clinical report forms to obtain socio-demographic data, medical history, and physical examination, dedicated questionnaires devoted to the evaluation of musculoskeletal manifestations and overall health are performed using a numerical rating scale – NRS for pain [17], fatigue [18] current disease activity (by patient and by physician), past disease activity [19] and global visual analogue scale [20] in addition to duration of morning stiffness evaluation [21]. In order to evaluate the health impact by different domains, the follow instruments are carried out, such as Health Assessment Questionnaire Disability Index (HAQ-DI) [22], 12-Item Short-Form Health Survey, (SF-12) and the EuroQol five-dimensional questionnaire (EQ-5D) [23], Work Productivity and Activity Impairment _ General Health (WPAI-GH) are performed to measure the effects of health in general and specific symptoms on work productivity and outside of work [24]. For mental health, anxiety and depression scales [25] are also investigated. Supplementary, a set of variables (core sets) in rheumatologic tools are meet, Clinical Disease Activity Index (27-CDAI), Simplified Disease Activity Index (27-SDAI) and Disease Activity Score 28 index [26] and The DN4 (Douleur Neuropathique 4 Questions), screening tool, used to indicate whether a patient may be suffering from neuropathic pain [27].

The Supplementary file presents the full contents of the clinical case report form as well as the questionnaires to be filled out at each study visit.

### Patient retention plan

Some strategies are implemented to avoid a percentage loss superior to 20% in our longitudinal cohorts such as barrier-reduction, community-building, follow-up/reminder, or tracing strategies. In order to enhance patient engagement, several activities are planned to be performed including patients, relatives and care partners, community and local health employers using social media, especially instagram, facebook, whatsapp. Confirm appointment and schedule visits by phone calls or phone messages, active search looking for local/regional patients in places where is known about the higher incidence of cases. Besides this, there is already a team in close contact with local health professionals, community leaders and the Municipal Health.

Department. All Sites have a close relationship with local stakeholders to improve engagement between the public and the site team, to contribute with the public healthcare system, and bring more information about awareness, prevention and treatment to a well known disease. In addition, as part of the Brazilian Health System, the Community Health Workers develop a door to door outreach program to take information and to connect the populations to other healthcare services to reach out patients and their community.

### Biological samples

**Fig. 3:**
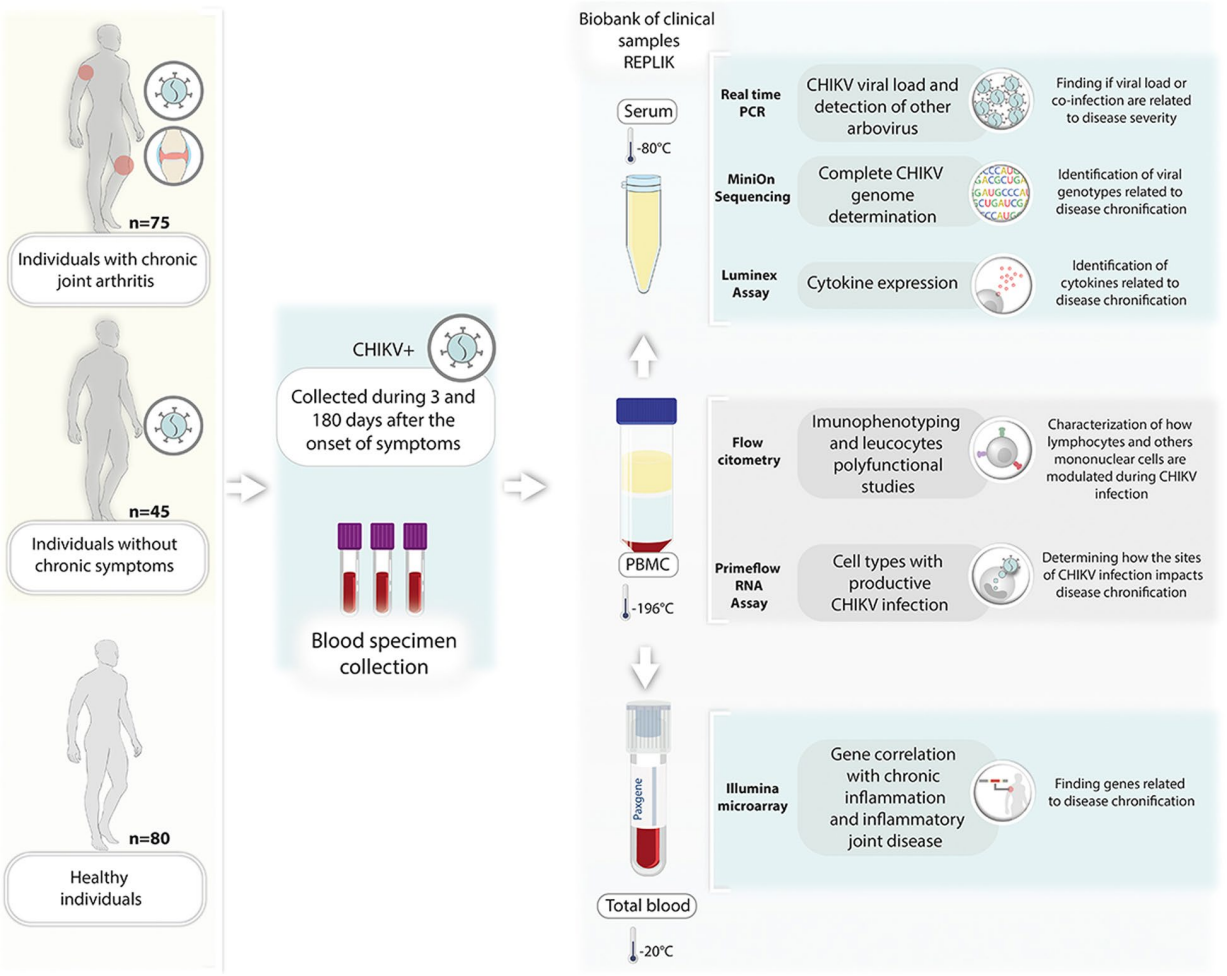
Biobank and Clinical samples scheame

Samples are collected in every study visit and include blood (i.e., whole blood, plasma, serum), urine, and saliva. Additional specimens such as synovial fluids and synovial biopsy material may be collected when the physician deems necessary and will be used only in the translational sub-studies. The collection procedure is performed by trained clinical staff. Figura3

### Biobank

Participants are asked to consent for their specimens to be maintained in a biobank and used in future research. Aliquots of total blood, serum, plasm, PAXgene, urine and peripheral blood mononuclear cells (PBMC) and when clinically indicated to be examined, synovial fluid, are stored at -80° C. The study has a central specimen bank in the coordination center, where samples collected from all regions are stored, and each site will have its local biorepository. The management and all activities involving these specimens, from sample collection to sample testing, are described in Standard Operating Procedure (SOP). A sharing platform and procedures are established to allow the use of such bioresources by members of the study teams or other researchers according to public health and scientific relevant criteria.

### Laboratory diagnosis

#### CHIKV polymerase chain reaction

EDTA-whole blood samples, urine, and saliva allow real-time polymerase chain reaction assay for the identification of CHIKV. Amplification assays are performed by qRT-PCR one step (Applied Biosystems), using the primers and probes described elsewhere [28]. In summary, qPCR are made with 2uL RNA (~ 100 ng), 2.5uL Primer and Probe Mix (IDT PrimeTime), and 12.5uL Master Mix (Applied Biosystems), which includes rhodamine as a passive reference. The RT-PCR reactions are performed at 48 °C for 30 min, and product amplification occurs by 45 cycles of denaturation at 95 oC for 15 s and annealing-extension at 60 oC for 1 min. Viral quantitation is performed based on the construction of a standard curve using decimal serial dilutions of a plasmid containing the target sequence.

### Study size power calculation

With 200 cases per site, analysis in a particular site will have 80% power and significance level of 0.05 to detect those individuals who will evolve to chronic CHIKV stage (Relative Risk: 50%) or are at risk of long-term sequelae. As we intend to perform analysis combining the cases from the different sites, we should have enough power to detect less stronger associations, even when considering loss to follow up and differences in enrollment per site due to diverse levels of CHIKV transmission per site.

### Pre-planned sub-studies

#### Genome sequencing and phylogenetic analysis

Samples strongly positive for CHIKV RNA (as judged by ct values between 20 to 30) and negative for other arboviruses (i.e., DENV, ZIKV, YF) will be further re-extracted and re-tested with a different qRT-PCR assay to confirm a molecular diagnosis. Subsequently, they will be selected for the Virome Capture Sequencing Platform for Vertebrate Viruses (VirCapSeq-VERT).

Total nucleic acid will be extracted from the approximately 200 ul volume of a clinical sample using the easyMAG automated platform (Biomerieux), following the manufacturer’s recommendations. Extracted nucleic acid will be eluted to a final volume of 50 uL in H2O. The samples with the lowest ct values will be enriched using the VirCapSeq-VERT protocol. Sequencing will be performed on the Illumina MiSeq platform (Illumina, San Diego, CA, USA) using a Reagent kit v3.

To investigate the origin of CHIKV isolated in the REPLICK network, the genomes generated and obtained via VirCapSeq and unbiased sequencing, will be included in the analysis with all the CHIKV genomes deposited in GenBank. This will result in a dataset containing a genomic sequence representing distinct viral genotypes. Then, sequences will be aligned using MAFFT, and viral phylogenies will be reconstructed using a maximum likelihood analysis implemented in RaxML. Analyses will be run for 108 generations, and convergence (sufficient sample size > 200) will be inspected using TRACER v1.6 after discarding 10% burn-in.

### Gene correlation with chronic inflammation and inflammatory joint disease

We will analyze gene populations for the search of associated genes using the base written in gene-wide and phenome-wide association chips through Illumina microarray, according to the technique specified by the manufacturer. As there is no previous data related to it, we will explore the research with molecular markers related to acute and chronic inflammation in CHIKV disease. We will use as our base two starting chips Infinium Omni 2.5–8 and Infinium QC Array-24.

### CHIKV persistence in different body fluids

#### Cytokines expression

Detection and quantification of cytokines in serum and synovial fluid will be performed by magnetic microbead immunoassay using a commercial Procarta human cytokine kit called cytokine 45-plex. In this assay we will measure the levels of GM-CSF, EGF, BDNF, bNGF, FGF-2, HGF, CCL2 (MCP-1), CCL3 (MIP-1alpha), MIP-1beta, CCL5 (RANTES), CXCL1 (GRO-alpha), CHCL12alpha (SDF-1alpha), CXCL10 (IP-10), Eotaxin, IFN-alpha, il-1ALPHA, il-1BETA, IL-1RA, IL-10, IL-13, IL-13, IL-15, IIL-17A, IL-18, IL-2, IL-21, IL-22, IL-23, IL-27, IL-31, IL-4, IL-5, IL-5, IL-6, IL-7, IL-8 (CXCL8), IL-12p70, LIF, Stem Cell Factor (SCF), TNF-alpha, VEGF-A, VEGF-D, PDGF-BB. And PIGF-1.

### Metabolome

The serum and synovial fluid collected will be analyzed by shotgun mass spectrometry and metabolites, including lipids, which present quantitative differences between the different samples that will be analyzed in silico using system biology tools. Predictive power will be determined using statistical tools such as PLS-DA.

### Lymphocyte polyfunctional studies and immunophenotyping

The cell suspension will be obtained from the total leukocyte population from plasma separation and cell pellet obtained from peripheral blood or synovial fluid. In this cell suspension, 5uL of monoclonal antibodies conjugated to different fluorochromes will be added. The following population will be analyzed in four tube assays: cell surface markers (CD3, CD4, CD8), T lymphocytes (CD5, CD8, CD10, CD14, CD19, CD45), and Rapa and lambda light chains f(AB’)2 (CD4, CD138, and CD16/56). After monoclonal antibodies are added, cells will be incubated in the dark for 15 min at room temperature and then analyzed by flow cytometry (Beckman Coulter Gallios). Isotope control antibodies will be used in this assay. At least 10.000 events will be acquired and analyzed using FlowJo software.

### Characterization by RNA flow of which cells harbor CHIKV virions

The characterization of cells harboring the CHIKV RNA in blood and synovial fluids will be determined by RNA flow assay through flow cytometry using commercially available kits, with genome probes and viral antigenome (prime-flow RNA Thermo assay). The cell subtypes will be delimited to T lymphocytes (CD45 + , CD3-, CD19-), B lymphocytes (CD45 + , CD3-, CD19 +), Natural killer cells (CD45 + , CD3-, CD19-, CD56 +), conventional dendritic cells (CD45 + , CD3-, CD19-, CD11c +), plasmacytoid dendritic cells (CD45 + , CD3-, CD19, CD123 +), and macrophages (CD45 + , CD68 +), then analyzed for the presence of the viral genome or antigenome. Compensation will be performed using UltraComp eBeads and PrimeFlow Compensation Kit (Affymetrix).

### Functional analysis of mononuclear cells and polymorphonuclear leukocytes

Peripheral blood mononuclear cells (MNC) and polymorphonuclear (PMN) leukocytes will be isolated by centrifugation using the Lymphoprep/Polymorphprep gradient. Red blood cells will be lysed, MNC, and PMNs will be isolated and washed in sterile saline and prepared for functional assays. The PMN responsiveness of CHIKV infected patients will be analyzed by different assays. Phagocytosis and microbicidal activity of these cells will be analyzed using *Escherichia coli* and *Staphylococcus aureus* according to established protocols. Oxidative metabolism of PMNs will be evaluated in vitro by measuring superoxide generation using chemiluminescent dyes (Luminol and Lucigenin). The constitutive and stimulated production of inflammatory mediators by PMNs will also be analyzed.

### Characterization of activation of macrophages, and dendritic cells by CHIKV

Monocytes obtained from blood will be differentiated in vitro to macrophages and dendritic cells, while neutrophils will be obtained directly by Ficoll-Paque gradient separation (GE Healthcare) and magnetic column selection according to the manufacturer’s instructions (EasySep Human Neutrophil Enrichment kit, StemCell Technologies). For differentiation of macrophages and dendritic cells, mononuclear cells will be isolated by positive immunomagnetic selection using anti-CD14 antibodies according to the manufacturer’s protocol (MACS, Miltenyi Biotec). The cells obtained will be grown in culture plates with RPMI medium supplemented with 10% fetal bovine serum (FBS), 1% penicillin–streptomycin and granulocytic colony-stimulated growth factor (M-CSF, 1X 104 U/ml) for differentiation macrophages, and GM-CSF and IL-4 (50 ng/mL) for differentiation in dendritic cells. All cultures will be kept at 37 C in a humidified atmosphere containing 5% CO2.

Isolated and differentiated cells will be infected with CHIKV. The culture supernatants will be collected at various periods after infection. The supernatant will be used to quantify inflammatory mediators, including IFN-gamma, TNF-alpha, TGF-beta, IL-10, IL-12, IL-1beta, and chemokines by ELISA according to the kit manufacturer (R&D Systems).

### Immunohistochemistry

We will perform histological analyses of synovial biopsies performed on individuals with CHIKV’s chronic arthritis. The tissue will be collected and fixed in 4% paraformaldehyde overnight at 4 C. the tissue will be then dehydrated, embedded in paraffin, and cut. The cut slides will be deparaffinized with serial xylol washes and rehydrated with decreasing absolute alcohol decreasing to 70% alcohol. Next, the section will be treated with a 0.1 M phosphate buffer pH 7.0 and treated with H2O2 for 10 min to block endogenous peroxidase and then incubated with blocking solution (Triton and skim milk) overnight. After this step, the tissues will be incubated for 1 h with the primary antibodies for CD123, CD11c, CD45, CD19, CD68, and CHIKV (monoclonal antibody 155,841). They will then be washed three times with 5% skimmed milk for 5 min and incubated for 40 min with the biotinylated secondary antibody (anti-mouse produced in goats – Molecular probes). After binding to a peroxide-conjugated streptavidin polymer, the development will be performed via the ABC kit (Vector).

### Data management

Data will be recorded using Redcap, and analyses will be performed with R software. The principal investigators from all the sites and those involved in data analysis will be given full access to the database. Data entry, validation, and query resolution will run in parallel with recruitment. No patient identifying information will be available during data analysis. Each study site will record study data pertaining to its study participants, however analyses will be performed by a statistician based in the coordination center. Individual records and patient identifiers (i.e. participant code number) will be kept confidential, accessible only to the local study team under the supervision of the study principal investigator. Laboratory staff only identify specimens by study ID, and will not have access to any personal identification information.

### Statistical analysis plan

The data from recruitment will be analyzed considering demographic characteristics of participants, presence of comorbidities, initial symptoms presentation (including days of symptoms, intensity of symptoms and medications used) as well as the values of the scores collected. The data will be summarized using simple summary statistics, such as percentages and frequencies for categorical variables and distributions of continuous variables examined with means, averages, medians and histograms as appropriate. As there is no defined criteria for chikungunya chronic disease and we aim to explore the diverse phenotypes that can be observed in chronically affected individuals, we aim to use persistence of pain with a score above 4 at the visual pain scale attributed by the participant as the primary outcome, while we will explore the other outcomes individually and in combination at distinct time points in order to try to establish a more well-defined criteria for categorizing intensity an type of chronic manifestation that could be used to guide management.

To evaluate how chronic manifestations of chikungunya affect physical and mental health, we will explore the scores and instruments applied with this aim and explore the association with risk factors related to demographic characteristics, initial presentation severity, virological and host immune aspects and type of interventions adopted using logistic and linear regression models. We also aim to evaluate the recovery rate of chikungunya using inverse propensity score weighted Cox Regression models at 30–90, 30–180, 30–360, 30–720 and 30–180 days from initial presentation. Further exploratory analyses will be performed for evaluation of clinically relevant predictive variables.

### Independent data monitoring committee

An independent data monitoring committee was established by the study management team and sponsors to ensure that the interests of patients taking part in the study are protected and that the scientific soundness of the study is kept from onset to completion. The committee is independent of the study team and free of significant conflicts of interest. The size of the committee ranges from 3–5 members of which two designated positions are a chair, who lead the deliberations and sign the official documents, and a statistician, to minimize subjective judgment.

### Ethics and confidentiality

The study is conducted following the protocol, the guidelines of the International Conference on Harmonization for Good Clinical Practice, the document of the Americas, and several resolution amendments approved by the Brazilian council of health. The study protocol was submitted and approved to the National IRB (CAAI: 07,936,919.8.1001.5262) and local IRB at each study site.

### Applicability of results

The results of this study will describe the burden of CHIKV in the Brazilian territory, providing evidence-based recommendations on the diagnosis and treatment of CHIKV infection.

### Communication plans

This study aims to address an indispensable communication demand in the healthcare system, creating effective communication between scientists and stakeholders, patients, participants, health professionals and society. Throughout the research, study results will be disseminated by the principal investigators and respective local teams. Meetings can be organized with the involvement of the study staff, participants, and community representatives. To make the results available to the intended audience, the content communicated by the research group has been divided by target groups: Researchers and team: (scientists, physicians, nurses, collaborators, community engagement professionals, laboratory personnel, project coordinators, administrative professionals, and others): offer basic communications training courses and standardize information across all research centers participating in this study. Both to be offered online through web conferences and/or in person. Participants and patients: promote biopsychosocial healthcare initiatives with participants, patients and/or relatives in the healthcare process of Chikungunya (diagnosys, treatment, and aftercare). Support groups monitored by an interdisciplinary team and community leaders; develop healthcare marketing campaigns. Society**:** develop multitargeted prevention healthcare communication pieces to be distributed at healthcare units, schools, events, others. The goal is to educate the population about the disease and the study, specially those living in at risk areas.

## Discussion

This study is the largest evaluation of the long-term sequelae of individuals infected with CHIKV in the Brazilian population focusing primarily on musculoskeletal manifestations, mental health, quality of life, and chronic pain. This study will also be the first to potentially identify risk groups and suggest changes in treatment regimens. The results obtained from this study will generate valuable data applicable for understanding the phenotypes and risk factors for chronic manifestation, thus helping to manage the burden of this viral disease.

Amongst the study limitations, it is important to mention the risk of loss to follow up and potential of participants noncompliance with study protocol. Results could also be biased, as chikungunya patients who are more affected by pain may be unable to attend the study appointments, or may be financially deprived, being unable to travel to the clinic. Thus, those who complete study follow-up may not well represent all chikungunya patients and a degree of selection bias may occur. We have included remote consultations and home visits as strategies to minimize this risk.

The study’s main strength is its comprehensive and broad evaluation of CHIKV-infected individuals using harmonized and standardized instruments. By using a prospective cohort and the ability to create nested cohorts or case–control analysis, and because Brazil is currently experiencing endemic and epidemic transmission of Chikungunya, a large sample size can be obtained which will strengthen the power and results of the study. Also, as this study is longitudinal over time and results are collected at regular time intervals, the risk of recall error is minimized.

Information about the project and its results will be disseminated to both public health and academic stakeholders.

This prospective cohort study will contribute to a better understanding of the disease and for the definition of management strategies that can make the patients' response more efficient. The results could be helpful not only in improving individual care but also in formulating appropriate public health policies and understanding the real impact of chronic disease on people's lives. It is important to highlight that most, if not all, of the recommendations published nationally and internationally are not substantiated in systematic analysis of the therapeutic response in a comparative way. This difficulty arises both from the epidemic nature of the infection, with no preparation time for trials to be defined, and from the complexity and diversity of pharmacological options, which can often be acquired by patients without a medical prescription. In this context, the health system needs quality information, collected systematically and with regional representation so that the aforementioned gaps can be filled in to support the strategies to be developed. The health, economic and psychosocial impact on CHIKV is high, as well as the burden on the health system, which needs to respond efficiently to this emergency. The proposed multicentric and multidisciplinary approach will allow the clinical characterization and natural history of arbovirus infection, mainly related to the involvement of the musculoskeletal system. Our approach also provides for the study of prognostic factors associated with severity and chronicity in addition to the evaluation of the therapeutic approach of patients with chronic pain due to CHIKV infection. In addition, these results will be very useful to help choose the better outcomes in chikungunya progression (specially between acute and chronic phases) to be performed in future trials. Thus, the results are expected to promote the production of evidence that substantiates the guidelines for management by the health system, with the prospect of a great impact on the health of the population.

## Supplementary Information

**Additional file 1.** REPLICK Case Report Form.

## Data Availability

The datasets generated and/or analyzed during the current study are available in the REPLICK repository, available at https://en.replick.net/replick.

## Abbreviation

CHIKV: Chikungunya virus
