# Supplementary material for: Multicenter study of the natural history and therapeutic responses of patients with chikungunya, focusing on acute and chronic musculoskeletal manifestations – a study protocol from the clinical and applied research in Chikungunya (REPLICK network)
Source: BMC Infect Dis. 2023 Jul 28;23:499. doi: 10.1186/s12879-023-08292-y (PMC10386654; doi:10.1186/s12879-023-08292-y)
Supplement: Supplementary file 1 — Additional file 1. REPLICK Case Report Form. [file 12879_2023_8292_MOESM1_ESM.pdf]

SOCIODEMOGRAPHIC DATA

Record ID ______________________________

CRF – Socio-demographic Information

Date of visit: ______________________________

Date of birth: ______________________________

Age ______________________________

Sex: ○ Female ○ Male

Point of care: ______________________________

Place of birth

State:

- ○ Acre Alagoas Amapá
- ○ Amazonas Bahia
- ○ Ceará Distrito Federal
- ○ Espírito Santo Goiás
- ○ Maranhão Mato Grosso
- ○ Mato Grosso do Sul Minas Gerais
- ○ Pará Paraíba Paraná
- ○ Pernambuco Piauí
- ○ Rio de Janeiro Rio Grande do Norte
- ○ Rio Grande do Sul Rondônia
- ○ Roraima Santa Catarina
- ○ São Paulo Sergipe
- ○ Tocantins

City: ______________________________

Country:

- Afghanistan
- South Africa
- Akrotiri
- Albania
- Germany
- Andorra
- Angola
- Anguila
- Antarctica
- Antígua and Barbuda
- Saudi Arabia
- Arctic Ocean
- Algeria
- Argentina
- Armenia
- Arruba
- Ashmore and Cartier Islands
- Atlantic Ocean
- Australia
- Áustria
- Azerbaijan
- Bahamas
- Bangladesh
- Barbados
- Bahrain
- Belgium
- Belize
- Benim
- Bermudas
- Bielorrussia
- Burma
- Bolivia
- Bosnia and Herzegovina
- Botswana
- Brazil
- Brunei
- Bulgaria
- Burkuina Faso
- Burundi
- Butan
- Cape Verde
- Camaroons
- Cambodia
- Canada
- Quatar
- Kazakhstan
- Chad
- Chile
- China
- Cyprus
- Clipperton Island
- Colombia
- Comoros
- Congo-Brazzaville
- Congo-Kinshasa
- Coral Sea Islands
- North Korea
- South Korea
- Ivory Coast
- Costa Rica
- Croacia
- Cuba
- Curacao
- Dhekelia
- Denmark
- Dominican Rep.
- Egypt
- United Arab Emirates
- Equador
- Eritrea
- Slovakia
- Slovenia
- Spain
- United States
- Estonia
- Ethiopia
- Faroe Islands
- Fiji
- Philippines
- Finland
- France
- Gabon
- Gambia
- Ghana
- Gaza Strip
- Georgia
- South Geórgia and South Sandwich
- Gibraltar
- Granada
- Greece
- Greenland
- Guam
- Guatemala
- Guernsey
- Guyana
- Guinea
- Equatorial Guinea
- Guinea Bissau
- Haiti
- Honduras
- Hong Kong
- Hungary
- Yemen
- Bouvet Is.
- Christmas Is.
- Norfolk Is.
- Cayman Is.
- Cook Is.
- Cocos Islands
- Falkland Is.
- Heard and McDonald
- Marshall Islands
- Solomon Islands
- Turks and Caicos Islands
- US Virgin Islands
- Ilhas Virgens Britânicas
- India
- Indian Ocean
- Indonesia
- Iran
- Iraq
- Ireland
- Island
- Israel
- Italy
- Jamaica
- Jan Mayen
- Japan
- Jersey
- Djibouti
- Jordan
- Kosovo
- Kuwait
- Laos
- Lesotho
- Latvia
- Lebanon
- Liberia
- Libya
- Liechtenstein
- Lithuania
- Luxembourg
- Macao
- Macedonia
- Madagascar
- Malaysia

○ Malawi ○ Maldives

REDCap

	Mali Malta Isle of<br>Northern Marianas Morocco<br>Mauritius Mauritania<br>Mexico Micronesia<br>Mozambique Moldavia<br>Monaco Mongolia<br>Monserrat Montenegro<br>Namibia<br>Nauru Navassa Island<br>Nepal Nicaragua<br>Niger Nigeria Niue<br>Norway New Caledonia<br>New Zealand Oman<br>Pacific Ocean Netherlands<br>Palau Panama Papua-New Guinea<br>Pakistan Paracel Islands<br>Paraguay Peru Pitcairn<br>French Polynesia Poland<br>Porto Rico Portugal<br>Kenya Kyrgyzstan<br>Kiribati UK<br>Central African Republic<br>Dominican<br>Czech Republic Republic<br>Romania<br>Rwanda Russia Salvador<br>Samoa American Samoa<br>Saint Helena St. Lucia<br>St. Bartholomew St. Kitts and<br>Neves San Marino<br>Saint Martin St. Pierre e Miquelon<br>São Tomé and Príncipe<br>St. Vincent and Grenadines<br>Western Sahara Seychelles<br>Senegal Sierra Leone<br>Serbia Singapore<br>Sint Maarten Syria<br>Somalia Southern Ocean<br>Spratly Islands Sri Lanka<br>Swaziland Sudan<br>South Sudan Sweden<br>Switzerland Suriname<br>Svalbard and Jan Mayen<br>Thailand Taiwan<br>Tajikistan Tanzania<br>British Indian Ocean Territory<br>French Southern Territories<br>East Timor Togo<br>Tokelau Tonga Trindade and<br>Tobago Tunisia Turkmenistan<br>Turkey Tuvalu Ucrânia<br>Uganda European Union<br>Uruguay Uzbekistan<br>Vanuatu Vatican City<br>Venezuela Vietnam<br>Wake Island Wallis and Futuna<br>West Bank Zambia<br>Zimbabwe
Skin color:	White Black Brown<br>Yellow Other
If other, state:	______________________________

Profession:	______________________________
Education Level:	○ Illiterate<br>○ Finished Elementary School<br>○ Did not finish Elementary School<br>○ Finished High School<br>○ Did not finish High School<br>○ Finished University Degree<br>○ Did not finish University Degree<br>○ Does not know or does not wish to answer
Marital Status:	○ Single ○ Married<br>○ Living together ○ Widow(er)<br>○ Separated<br>○ Divorced

HISTORY OF MEDICATION

CRF - Medication History

Have you taken any medication in the last 15 days? ○ Yes ○ No

How many different medicines?
○ 1
○ 2
○ 3
○ 4
○ 5
○ 6
○ 7
○ 8
○ 9
○ 10

Medication 1

Generic Name: ______________________

Amount (mg/mL): ______________________

Dosage: ______________________

Duration of Treatment (days): ______________________

Therapeutic Indication: ______________________

Medication prescribed by doctor or dentist: ○ Yes ○ No

How many days did the participant take this medicine in the last two weeks? ______________________

Did the participant present proof of use of the medication? ○ Yes ○ No (Packaging, package insert, prescription)

Medication 2

Generic Name: ______________________

Amount (mg/mL): ______________________

Dosage: ______________________

Duration of Treatment (days): __________________________________

Therapeutic Indication: __________________________________

Medication prescribed by doctor or dentist: ○ Yes ○ No

How many days did the participant take this medicine in the last two weeks? __________________________________

Did the participant present proof of use of the medication? ○ Yes ○ No
(Packaging, package insert, prescription)

Medication 3

Generic Name: __________________________________

Amount (mg/mL): __________________________________

Dosage: __________________________________

Duration of Treatment (days): __________________________________

Therapeutic Indication: __________________________________

Medication prescribed by doctor or dentist: ○ Yes ○ No

How many days did the participant take this medicine in the last two weeks? __________________________________

Did the participant present proof of use of the medication? ○ Yes ○ No
(Packaging, package insert, prescription)

Medication 4

Generic Name: __________________________________

Amount (mg/mL): __________________________________

Dosage: __________________________________

Duration of Treatment (days): __________________________________

Therapeutic Indication: __________________________________

Medication prescribed by doctor or dentist:	○ Yes ○ No
How many days did the participant take this medicine in the last two weeks?	__________________________
Did the participant present proof of use of the medication?	○ Yes ○ No<br>(Packaging, package insert, prescription)

Medication 5

Generic Name:	__________________________
Amount (mg/mL):	__________________________
Dosage:	__________________________
Duration of Treatment (days):	__________________________
Therapeutic Indication:	__________________________
Medication prescribed by doctor or dentist:	○ Yes ○ No
How many days did the participant take this medicine in the last two weeks?	__________________________
Did the participant present proof of use of the medication?	○ Yes ○ No<br>(Packaging, package insert, prescription)

Medication 6

Generic Name:	__________________________
Amount (mg/mL):	__________________________
Dosage:	__________________________
Duration of Treatment (days):	__________________________
Therapeutic Indication:	__________________________
Medication prescribed by doctor or dentist:	○ Yes ○ No
Did the participant present proof of use of the medication?	__________________________

Did the participant present proof of use of the medication?	◯ Yes ◯ No<br>(Packaging, package insert, prescription)

Medication 7

Generic Name:	__________________________
Amount (mg/mL):	__________________________
Dosage:	__________________________
Duration of Treatment (days):	__________________________
Therapeutic Indication:	__________________________
Medication prescribed by doctor or dentist:	◯ Yes ◯ No
How many days did the participant take this medicine in the last two weeks?	__________________________
Did the participant present proof of use of the medication?	◯ Yes ◯ No<br>(Packaging, package insert, prescription)

Medication 8

Generic Name:	__________________________
Amount (mg/mL):	__________________________
Dosage:	__________________________
Duration of Treatment (days):	__________________________
Therapeutic Indication:	__________________________
Medication prescribed by doctor or dentist:	◯ Yes ◯ No
How many days did the participant take this medicine in the last two weeks?	__________________________
Did the participant present proof of use of the medication?	◯ Yes ◯ No<br>(Packaging, package insert, prescription)

Medication 9

Generic Name:	__________________
Amount (mg/mL):	__________________
Dosage:	__________________
Duration of Treatment (days):	__________________
Therapeutic Indication:	__________________
Medication prescribed by doctor or dentist:	○ Yes ○ No
How many days did the participant take this medicine in the last two weeks?	__________________
Did the participant present proof of use of the medication?	○ Yes ○ No<br>(Packaging, package insert, prescription)

Medication 10

Generic Name:	__________________
Amount (mg/mL):	__________________
Dosage:	__________________
Duration of Treatment (days):	__________________
Therapeutic Indication:	__________________
Medication prescribed by doctor or dentist:	○ Yes ○ No
How many days did the participant take this medicine in the last two weeks?	__________________
Did the participant present proof of use of the medication?	○ Yes ○ No<br>(Packaging, package insert, prescription)

Epidemiological History

CRF - Epidemiological History

Did the patient travel recently (in the last 30 days)?	○ Yes ○ No
To which State:	○ Acre ○ Alagoas ○ Amapá ○ Amazonas ○ Bahia ○ Ceará ○ Distrito Federal ○ Espírito Santo ○ Goiás ○ Maranhão ○ Mato Grosso ○ Mato Grosso do Sul ○ Minas Gerais ○ Pará ○ Paraíba ○ Paraná ○ Pernambuco ○ Piauí ○ Rio de Janeiro ○ Rio Grande do Norte ○ Rio Grande do Sul ○ Rondônia ○ Roraima ○ Santa Catarina ○ São Paulo ○ Sergipe ○ Tocantins
City/Town:	______________________________
Day of return:	○ Not known ○ 1 ○ 2 ○ 3 ○ 4 ○ 5 ○ 6 ○ 7 ○ 8 ○ 9 ○ 10 ○ 11 ○ 12 ○ 13 ○ 14 ○ 15 ○ 16 ○ 17 ○ 18 ○ 19 ○ 20 ○ 21 ○ 22 ○ 23 ○ 24 ○ 25 ○ 26 ○ 27 ○ 28 ○ 29 ○ 30 ○ 31
Month of return:	○ Not known ○ Jan ○ Feb ○ Mar ○ Apr ○ May ○ Jun ○ Jul ○ Aug ○ Sep ○ Oct ○ Nov ○ Dec
Year of return:	______________________________
Length of stay in the city/town:	______________________________ (in days)
Are there any cases of Dengue where you live/work?	○ Yes ○ No ○ Not known
Are there any cases of Zika where you live/work?	○ Yes ○ No ○ Not known
Are there any cases of Chikungunya where you live?	○ Yes ○ No ○ Not known

Are there any cases of Chikungunya where you work?	○ Yes ○ No ○ Not known
Do you live or work near wasteland?	○ Yes ○ No
History of Dengue	○ Yes ○ No ○ Not known
Month:	○ Not known ○ Jan<br>○ Feb ○ Mar ○ Apr<br>○ May ○ Jun ○ Jul<br>○ Aug ○ Sep ○ Oct<br>○ Nov ○ Dec
Year:	____________________
History of Zika	○ Yes ○ No ○ Not known
Month:	○ Not known ○ Jan<br>○ Feb ○ Mar ○ Apr<br>○ May ○ Jun ○ Jul<br>○ Aug ○ Sep ○ Oct<br>○ Nov ○ Dec
Year:	____________________

Vaccination History

Have you been vaccinated against Yellow Fever (last 10 years)?	○ Yes ○ No ○ Not known
Year:	____________________
Have you been vaccinated against Dengue?	○ Yes ○ No ○ Not known
Year:	____________________

PREVIOUS MEDICAL HISTORY

**CRF – Previous Medical History (Comorbidities)**	
Do you have any comorbidities?	○ Yes ○ No
Anemia:	○ Yes ○ No ○ Not known
Diabetes:	○ Yes ○ No ○ Not known
Systematic arterial hypertension:	○ Yes ○ No ○ Not known
Asthma:	○ Yes ○ No ○ Not known
Rhinitis:	○ Yes ○ No ○ Not known
Chronic obstructive pulmonary disease (COPD):	○ Yes ○ No ○ Not known
Coronary disease:	○ Yes ○ No ○ Not known
Kidney disease:	○ Yes ○ No ○ Not known
Please specify:	__________________________________
Rheumatism:	○ Yes ○ No ○ Not known
Please specify:	__________________________________
Vascular disease:	○ Yes ○ No ○ Not known
Please specify:	__________________________________
Benign, malignant or nonspecific neoplasms:	○ Yes ○ No ○ Not known
Please specify:	__________________________________
Liver Disease:	○ Yes ○ No ○ Not known
Please specify:	__________________________________
HIV infection:	○ Yes ○ No ○ Not known
Other Relevant Comorbidities:	○ Yes ○ No
Which:	__________________________________

CLINICAL EVALUATION

CRF – Clinical Evaluation

Scheduled visit nº: __________________________________

Date of visit: __________________________________

Participant ID: __________________________________

Participant QR Code: __________________________________

Vital Signs

Weight (kg): __________________________________

Height (cm): __________________________________

BMI: __________________________________

Blood pressure (mmHg):

Systolic: __________________________________

Diastolic: __________________________________

Heart rate (bpm): __________________________________

Respiratory frequency (rpm): __________________________________

Body temperature (ºC): __________________________________

Physical exam

Diagnostic impression:

Prescription Drugs

Has medication been prescribed? ○ Yes ○ No

How many different medicines?
○ 1
○ 2
○ 3
○ 4
○ 5
○ 6
○ 7
○ 8
○ 9
○ 10

Medication 1:

Generic Name: __________________________________

Amount (mg/mL): __________________________________
(Unit)

Dosage: __________________________________

Duration of Treatment (days): __________________________________

Therapeutic Indication: __________________________________

Prescription Drugs

Medication 2:

Nome Genérico: __________________________________

Amount (mg/mL): __________________________________
(Unit)

Dosage: __________________________________

Duration of Treatment (days): __________________________________

Therapeutic Indication: __________________________________

Prescription Drugs

Medication 3:

Generic Name: __________________________________

Amount (mg/mL): __________________________________
(Unit)

Dosage: __________________________________

Duration of Treatment (days): __________________________________

Therapeutic Indication: __________________________________

Prescription Drugs

Medication 4:

Generic Name: __________________________________

Amount (mg/mL): __________________________________
(Unit)

Dosage: __________________________________

Duration of Treatment (days): __________________________________

Therapeutic Indication: __________________________________

Prescription Drugs

Medication 5:

Generic Name: __________________________________

Amount (mg/mL): __________________________________
(Unit)

Dosage: ______________________

Duration of Treatment (days): ______________________

Therapeutic Indication: ______________________

Prescription Drugs

Medication 6:

Generic Name: ______________________

Amount (mg/mL): ______________________
(Unit)

Dosage: ______________________

Duration of Treatment (days): ______________________

Therapeutic Indication: ______________________

Prescription Drugs

Medication 7:

Generic Name: ______________________

Amount (mg/mL): ______________________
(Unit)

Dosage: ______________________

Duration of Treatment (days): ______________________

Therapeutic Indication: ______________________

Prescription Drugs

Medication 8:

Generic Name: __________________________________

Amount (mg/mL): __________________________________
(Unit)

Dosage: __________________________________

Duration of Treatment (days): __________________________________

Therapeutic Indication: __________________________________

Prescription Drugs

Medication 9:

Generic Name: __________________________________

Amount (mg/mL): __________________________________
(Unit)

Dosage: __________________________________

Duration of Treatment (days): __________________________________

Therapeutic Indication: __________________________________

Prescription Drugs

Medication 10:

Generic Name: __________________________________

Amount (mg/mL): __________________________________
(Unit)

Dosage: __________________________________

Duration of Treatment (days): __________________________________

Therapeutic Indication: ______________________

RHEUMATOLOGICAL CONSULTATION

CRF - Rheumatological Consultation

Date: ______________________________

Diagnostic hypothesis: ○ Dengue ○ Zika ○ Chikungunya

How long has patient had musculoskeletal manifestations? ______________________________

Prior rheumatological condition: ○ No ○ Yes

Which? ______________________________

	0	1	2	3	4	5	6	7	8	9	10
Pain:	○	○	○	○	○	○	○	○	○	○	○
Fatigue:	○	○	○	○	○	○	○	○	○	○	○
Global Health Evaluation:	○	○	○	○	○	○	○	○	○	○	○
Disease Activity (Patient):	○	○	○	○	○	○	○	○	○	○	○
Disease Activity (Doctor):	○	○	○	○	○	○	○	○	○	○	○

Pain: ______________________________

Edema: ______________________________

According to Homunculus, considering 44 total joints:

Number of Painful Joints - NPJ: ______________________________

Number of Swollen Joints - NSJ: ______________________________

Bursitis: ○ Yes ○ No

Region: ______________________________

Tenosynovitis: ○ Yes ○ No

Region: ______________________________

Enthesitis: ○ Yes ○ No

Region: ______________________

Carpal Tunnel Syndrome: ○ Yes ○ No

Region: ______________________

Other: ○ Yes ○ No

Region: ______________________

Rheumatoid Arthritis Disease Activity Index: ______________________

	None	Mild	Moderate	Severe
Neck:	○	○	○	○
Back:	○	○	○	○

Left Side

	None	Mild	Moderate	Severe
Shoulder:				
Elbow:	○	○	○	○
Wrist:	○	○	○	○
Hand:	○	○	○	○
Hip:	○	○	○	○
Knee:	○	○	○	○
Ankle:	○	○	○	○
Foot:	○	○	○	○

Right Side

	None	Mild	Moderate	Severe
Shoulder:	○	○	○	○
Elbow:	○	○	○	○
Wrist:	○	○	○	○
Hand:	○	○	○	○
Hip:	○	○	○	○
Knee:	○	○	○	○
Ankle:	○	○	○	○
Foot:	○	○	○	○

DAS-28 Score for Rheumatoid Arthritis:
Use only 28 joints (shoulders, elbows, wrists, metacarpophalangeal joints, proximal interphalangeal joints, and knees, bilaterally)

ESR: ______________________ (mm/hr)

DAS-28ESR: ______________________

Remission

Low

Moderate

High

Clinical Disease Activity Index - CDAI

CDAI: ______________________

Remission

Low

Moderate

High

Clinical Disease Activity Index - SDAI

C-reactive protein: ______________________ (mg/dL)

SDAI: ______________________

Remission

Low

Moderate

High

Patient with disease activity classified as: ○ Remission ○ Low ○ Moderate ○ High

SELF-APPLICABLE QUESTIONNAIRES

Scheduled visit nº: ______________________

Date: ______________________

Phase of study:
- ◯ Acute Phase
- ◯ Chronic Phase
- ◯ Sub-acute Stage

Numerical scales to be filled in by patient

	0	1	2	3	4	5	6	7	8	9	10
Pain: How much joint pain have you had in the last week?<br>0- No pain<br>10- Worst pain possible	◯	◯	◯	◯	◯	◯	◯	◯	◯	◯	◯
Fatigue: How much abnormal tiredness has been a problem for you in the last week?<br>0- Fatigue is not a problem<br>10- Fatigue is a big problem	◯	◯	◯	◯	◯	◯	◯	◯	◯	◯	◯
Disease activity today: Considering the pain and swelling of your articulations, how would you describe the activity of your rheumatic condition today?<br>0- No activity<br>10- Extremely active	◯	◯	◯	◯	◯	◯	◯	◯	◯	◯	◯
Disease activity in the last 6 months: Considering the pain and swelling of your articulations, how would you describe the the activity of your rheumatic condition in the last six months?<br>0- No activity<br>10- Extremely active	◯	◯	◯	◯	◯	◯	◯	◯	◯	◯	◯
General Health: Considering all the manners in which your health and disease affect you right now, how do you feel?<br>0- Very well<br>10- Very bad	◯	◯	◯	◯	◯	◯	◯	◯	◯	◯	◯

Morning Stiffness: Were your joints stiff when you woke up today?

- ◯ No
- ◯ < 30min
- ◯ > 30min and < 1 h
- ◯ > 1 and < 2 hrs
- ◯ > 2 and < 4 hrs
- ◯ > 4 hrs
- ◯ All day

HAQ (Health Assessment Questionnaire

In this section, we would like to know how your illness affects your ability to perform your daily activities.

Please mark the answer that best describes your ability to perform day to day activities. IN THE LAST WEEK:

	No difficulty	Some difficulty	A lot of difficulty	Unable to do them
1. Dressing, including tying shoelaces and buttoning clothes	◯	◯	◯	◯
2. Washing your hair	◯	◯	◯	◯
3. Getting up from a chair without help	◯	◯	◯	◯
4. Lying down or getting out of bed	◯	◯	◯	◯
5. Cutting meat	◯	◯	◯	◯
6. Raising a mug or glass to your mouth	◯	◯	◯	◯
7. Opening a carton of milk	◯	◯	◯	◯
8. Walking outside on a flat surface	◯	◯	◯	◯
9. Climb 5 steps	◯	◯	◯	◯
10. Washing and drying your body	◯	◯	◯	◯
11. Having a bath / shower	◯	◯	◯	◯
12. Sitting on or getting up from the toilet	◯	◯	◯	◯
13. Reaching and getting a 2 kg object (for example, a sack of potatoes) from above your head	◯	◯	◯	◯
14. Bend over or crouch to pick up clothes from the floor	◯	◯	◯	◯
15. Open a car door	◯	◯	◯	◯

16. Open pots that have previously been opened	○	○	○	○
17. Open and close taps	○	○	○	○
18. Go to the bank and go shopping	○	○	○	○
19. Get in and out of a car	○	○	○	○
20. Do housework (for example, sweeping and working in the garden)	○	○	○	○

SF-12 (Short Form-12)

In this section, this questionnaire asks some questions about the activities you perform during your day. Answer each question by marking the answer as indicated. If you are unsure how to respond, please try to answer as best you can.

1. In general would you say your health is:

○ Excellent ○ Very good ○ Good ○ Bad ○ Very bad

The following items are about activities you could currently do during an ordinary day. Because of your health, would you have difficulty doing these activities? In this case, how much difficulty?

2. Moderate activities such as moving a table, vacuuming, playing ball, sweeping the house:

○ My disease makes it very difficult ○ My disease makes it a little difficult ○ It doesn't make it difficult at all

3. Climb various flights of stairs

○ My disease makes it very difficult ○ My disease makes it a little difficult ○ It doesn't make it difficult at all

During the past four weeks, have you had any of the following problems with your work or regular daily activity as a result of your physical health?

4. Did you perform fewer tasks than you would have liked? ○ Yes ○ No

5. Have you been limited in your type of work or in other activities? ○ Yes ○ No

During the past four weeks, have you had any of the following problems with your work or regular daily activity as a result of an emotional problem (such as feeling depressed or anxious)?

6. Did you perform fewer tasks than you would have liked? ○ Yes ○ No

7. Haven't worked or done any of the activities as carefully as you usually do? ○ Yes ○ No

8. During the past four weeks, how much has the pain interfered with your normal work (including both work, away from home and indoors)?

○ Not at all ○ A little ○ Moderately ○ A lot ○ Extremely

	All the time	Very frequently	Frequently	Sometimes	Not often	Never
9. How often do you feel calm or relaxed?	○	○	○	○	○	○
10. How often do you feel energetic?	○	○	○	○	○	○
11. How often do you feel depressed or down?	○	○	○	○	○	○

12. During the past four weeks, how much of your time has your physical health or your emotional problems interfered with your social activities such as visiting friends, relatives, etc.)?

○ The whole time ○ Most of the time
○ Some of the time
○ A little of the time
○ None of the time

Hospital Anxiety and Depression Questionnaire (HAD a/d)

This questionnaire will help your doctor know how you are feeling. Read all the sentences. Mark with an X how you were feeling LAST WEEK. No need to think too much about each question. In this questionnaire, spontaneous answers have more value than those that are thought too much about. Mark only one answer for each question.

1. I feel tense or stressed:

○ Very often ○ Often ○ Sometimes ○ Never

2. I still like the same things as before:

○ Yes, just as before ○ Not as much as before ○ Just a little ○ I don't feel pleasure anymore

3. I feel a kind of fear, as if something bad is going to happen:

○ Yes, and quite strongly ○ Yes, but not very strongly ○ A little, but I'm not worried
○ Not at all

4. I laugh and have fun when I see some funny things:

○ Just as before ○ Nowadays, a bit like before ○ Nowadays, a lot less
○ I don't laugh anymore

5. I have a head full of worries:

○ Very often ○ Often ○ Sometimes ○ Rarely

6. I feel happy:

○ Never ○ Rarely ○ Often ○ Very often

7. I can sit at ease and feel relaxed:

○ Yes, almost always ○ Often ○ Not often ○ Never

8. I am slow to think and do things:

○ Almost always ○ Very often ○ Sometimes ○ Never

9. I have a strong feeling of fear, like a cold feeling in my belly or a tightness in my stomach:

○ Never ○ Sometimes ○ Very often ○ Almost always

10. I have lost interest in taking care of my appearance:

○ Completely ○ I'm not taking care of myself anymore like I should ○ Maybe not as much as before
○ I take care of myself the same way as before

11. I feel restless, as if I can't stand still wherever I go:

○ Yes, a lot ○ Quite a lot ○ A little ○ I don't feel that way

12. I'm looking forward to the good things yet to come:

○ Just like before ○ A little less than before ○ A lot less than before ○ Almost never

13. I suddenly have a feeling of panic:

○ Almost the whole time ○ Often ○ Sometimes ○ I don't suffer from that

14. When I watch a good TV show, radio show or when I read something I can enjoy it:

○ Almost always ○ Often ○ Not very often ○ Almost never

WPAI (Work Productivity and Activity Impairment)

The questions below ask about the effect of your health problems on your ability to work and perform activities regularly.
By health problems we are referring to any physical or emotional problem or symptom.

1) Are you currently employed (paid work)? ○ Yes ○ No

The next questions refer to the last seven days, not including today.

2) During the past seven days, how many hours did you have to stop working because of your health problems?

____

(Include hours not worked when you were sick, late, left early, etc., because of your health or digestive problems. Do not include the time you missed in order to participate in this study.)

3) During the past seven days, how many hours did you stop working for any other reason, such as vacations, holidays, free time to participate in this study?

____

4) During the last seven days, how many hours did you work?

____

(If "0", write "0")

5) During the past seven days, how much did your health problems affect your productivity while you were working? Think about the days you were limited in the amount or type of work you could do, the days you did less than you would have liked to do, or the days you were less careful than usual in your work.
If health problems have affected your work only slightly, choose a low number. Choose a high number if health problems have affected your work a lot.

0- Health problems did NOT affect my work
10- Health problems completely prevented me from working

○ 0 ○ 1 ○ 2 ○ 3 ○ 4 ○ 5 ○ 6 ○ 7 ○ 8 ○ 9 ○ 10

6) During the past seven days, how much have your health problems affected your ability to do your regular daily activities, (other than work at your job)? By regular activities we mean common activities you do at home, shopping, childcare, working out, study etc. Think about the times you were limited in the amount or type of activities you could do and the times you did less than you would have liked. If health problems have affected your activities only slightly, choose a low number. Choose a high number if health problems have affected your activities a lot.

0- Health problems did NOT affect my regular daily activities
10- Health problems completely prevented me from doing regular daily activities

○ 0 ○ 1 ○ 2 ○ 3 ○ 4 ○ 5 ○ 6 ○ 7 ○ 8 ○ 9 ○ 10

RADAI (Rheumatoid Arthritis Disease Activity Index)

	None	Mild	Moderate	Severe
Neck	○	○	○	○
Back	○	○	○	○

Left side

	None	Mild	Moderate	Severe
Shoulder	◯	◯	◯	◯
Elbow	◯	◯	◯	◯
Wrist	◯	◯	◯	◯
Hand	◯	◯	◯	◯
Hip	◯	◯	◯	◯
Knee	◯	◯	◯	◯
Ankle	◯	◯	◯	◯
Foot	◯	◯	◯	◯

Right side

	None	Mild	Moderate	Severe
Shoulder	◯	◯	◯	◯
Elbow	◯	◯	◯	◯
Wrist	◯	◯	◯	◯
Hand	◯	◯	◯	◯
Hip	◯	◯	◯	◯
Knee	◯	◯	◯	◯
Ankle	◯	◯	◯	◯
Foot	◯	◯	◯	◯

Neuropathic Pain Questionnaire - DN-4

Does your pain have one or more of the following characteristics?

	Yes	No
1. Burning:	◯	◯
2. Cold Feeling:	◯	◯
3. Electric shock:	◯	◯

Is 1 or more of the following symptoms present in the areas where you feel pain?

	Yes	No
4. Tingling:	◯	◯
5. Pinpricks:	◯	◯
6. Numbness:	◯	◯
7. Itching:	◯	◯

Questions completed via medical examination

Pain is located in an area where physical examination may reveal one or more of the following characteristics:

	Yes	No
8. Hypoesthesia when touched:	◯	◯
9. Hypoesthesia when stung by a bee	◯	◯

In the painful area the pain may be caused or increased by:

	Yes	No
10. Brushing:	◯	◯

Check which statement best describes your state of health TODAY

Descriptive System EQ-5D-3L

Mobility:
◯ I have no problem walking
◯ I have some problems walking
◯ I am bedridden

Personal care:
◯ I have no problems with my personal care
◯ I have some problems washing and dressing
◯ I am incapable

Usual Activities:
◯ I have no problem performing my usual activities
◯ I have some problems performing my usual activities
◯ I am unable to perform my usual activities
(e.g. work, study, housework, family or leisure activities)

Pain/ Feeling unwell:
◯ I have no pain or discomfort
◯ I have moderate pain or discomfort
◯ I have extreme pain or discomfort

Anxiety/Depression:
◯ I'm not anxious or depressed
◯ I am moderately anxious or depressed
◯ I am extremely anxious or depressed

Visual Analog Scale (VAS):
0 50 100

(Place a mark on the scale above)

IMAGING EXAMS

Scheduled visit nº: ______________________________

PID Number: ______________________________

Date: ______________________________

Was an imaging exam performed? ○ Yes ○ No

Motive: ______________________________

Type of exam:
○ Ultrasound
○ X-ray
○ Tomography
○ MR scan
○ Other

Which? ______________________________

Date of test: ______________________________

Region/Area ______________________________

Result: ○ Normal ○ Abormal ○ Not known

Description of the abnormality

Type: ______________________________

Location: ______________________________

Size: ______________________________

Attachments

Report atttached:	○ Yes ○ No
Attach image:	
Images uploaded:	○ Yes ○ No
Attach image:	

LABORATORY RESULTS

PID Number: ______________________

Scheduled visit nº: ______________________

Date: ______________________

BLOOD

Blood test done? ○ Yes ○ No

Motive: ______________________

Date blood test done: ______________________

Time blood test done: ______________________
(HH:MM)

Blood count done? ○ Yes ○ No

Motive: ______________________

Date blood count done: ______________________

Hemoglobin ○ Not known /Error

Result: ______________________

Unit of measurement: ○ g/L ○ g/dL ○ Other

Other: ______________________

Hematocrit ○ Not known /Error

Result: ______________________

Unit of measurement: ○ % ○ Other

Other: ______________________

Red blood cells	○ Not known /Error
Result:	______________________
Unit of measurement:	○ Millions/mm³ ○ Millions/μL ○ Other
Other:	______________________
MCV	○ Not known /Error
Result:	______________________
Unit of measurement:	○ fL ○ μm³ ○ u3 ○ Other
Other:	______________________
MCH	○ Not done ○ Not known/Error
Result:	______________________
Unit of measurement:	○ pg ○ Other
Other:	______________________
MCHC	○ Not done ○ Not known /Error
Result:	______________________
Unit of measurement:	○ % ○ g/dL ○ Other
Other:	______________________
White blood cell count	○ Not known /Error
Result:	______________________
Unit of measurement:	○ mm³ ○ x10³/μl ○ Other
Other:	______________________
Segmented nucleus	○ Not known /Error

Result:	______________________
Unit of measurement:	○ mm³ ○ % ○ Other
Other:	______________________
Band cell	○ Not known /Error
Result:	______________________
Unit of measurement:	○ mm³ ○ % ○ Other
Other:	______________________
Neutrophils	○ Not done ○ Not known /Error
Result:	______________________
Unit of measurement:	○ mm³ ○ % ○ Other
Other:	______________________
Metamelocytes	○ Not done ○ Not known /Error
Result:	______________________
Unit of measurement:	○ mm³ ○ % ○ Other
Other:	______________________
Lymphocytes	○ Not done ○ Not known /Error
Result:	______________________
Unit of measurement:	○ mm³ ○ % ○ Other
Other:	______________________
Monocytes	○ Not done ○ Not known /Error
Result:	______________________

Unit of measurement:	○ mm³ ○ % ○ Other
Other:	__________________________
Eosinophils	○ Not done ○ Not known /Error
Result:	__________________________
Unit of measurement:	○ mm³ ○ % ○ Other
Other:	__________________________
Basophils	○ Not done ○ Not known /Error
Result:	__________________________
Unit of measurement:	○ mm³ ○ % ○ Other
Other:	__________________________
Platelet count	○ Not done ○ Not known /Error
Result:	__________________________
Unit of measurement:	○ mm³ ○ mil/mm³ ○ Other
Other:	__________________________
ERS	○ Not done ○ Not known /Error
Result:	__________________________
Unit of measurement:	○ mm/h ○ Other
Other:	__________________________

Biochemistry

Was biochemistry performed?	○ Yes ○ No
Date performed	__________________________________
Motive:	__________________________________
AST	○ Not done ○ Not known /Error
Result:	__________________________________
Unit of measurement:	○ U/L ○ IU/L ○ Other
Other:	__________________________________
ALT	○ Not done ○ Not known /Error
Result:	__________________________________
Unit of measurement:	○ U/L ○ IU/L ○ Other
Other:	__________________________________
Glucose	○ Not done ○ Not known /Error
Result:	__________________________________
Unit of measurement:	○ mg/dL ○ Other
Other:	__________________________________
Urea	○ Not done ○ Not known /Error
Result:	__________________________________
Unit of measurement:	○ mg/dL ○ Other
Other:	__________________________________
Creatinine	○ Not done ○ Not known /Error
Result:	__________________________________

Unit of measurement:	○ mg/dL ○ Other
Other:	__________________________________
C-reactive protein	○ Not done ○ Not known /Error
Result:	__________________________________
Unit of measurement:	○ mg/dL ○ Other
Other:	__________________________________
Were other tests done?	○ Yes ○ No
How many?	○ 1 ○ 2 ○ 3 ○ 4
Test:	__________________________________
Result:	__________________________________
Unit of measurement:	__________________________________
Test:	__________________________________
Result:	__________________________________
Unit of measurement:	__________________________________
Test:	__________________________________
Result:	__________________________________
Unit of measurement:	__________________________________
Test:	__________________________________
Result:	__________________________________
Unit of measurement:	__________________________________

Serological test: DPP Dengue/Zika/Chikungunya

Was a serological test performed?	○ Yes ○ No
Date of test:	_______________
Motive:	_______________
Dengue	
Dengue IgG	○ IgG reactive ○ IgG non-reactive ○ IgG indeterminado
Dengue IgM	○ IgM reactive ○ IgM non-reactive ○ IgM undetermined
Zika	
Zika IgG	○ IgG reactive ○ IgG non-reactive ○ IgG undetermined
Zika IgM	○ IgM reactive ○ IgM non-reactive ○ IgM undetermined
Chikungunya	
Chikungunya IgG	○ IgG reactive ○ IgG non-reactive ○ IgG undetermined
Chikungunya IgM	○ IgM reactive ○ IgM non-reactive ○ IgM undetermined

Molecular test: ZDC Dengue/Zika/Chikungunya - SANGUE

Molecular test done?	○ Yes ○ No
Date of test	_______________
Motive:	_______________
Dengue	○ Detected ○ Not detected ○ Inconclusive
Ct:	_______________
Zika	○ Detected ○ Not detected ○ Inconclusive
Ct:	_______________

Chikungunya	○ Detected ○ Not detected ○ Inconclusive
Ct:	__________________

URINE

Was a urine sample taken?	○ Yes ○ No
Date taken:	__________________
Time taken:	__________________ (HH:MM)
Motive:	__________________

Molecular test: ZDC Dengue/Zika/Chikungunya - URINE

Molecular test performed?	○ Yes ○ No
Date test performed:	__________________
Motive:	__________________
Dengue	○ Detected ○ Not detected ○ Inconclusive
Ct:	__________________
Zika	○ Detected ○ Not detected ○ Inconclusive
Ct:	__________________
Chikungunya	○ Detected ○ Not detected ○ Inconclusive
Ct:	__________________

SALIVA

Was a saliva sample taken? ○ Yes ○ No

Date taken ______________________________

Time taken ______________________________
(HH:MM)

Motive: ______________________________

Molecular test: ZDC Dengue/Zika/Chikungunya - SALIVA

Molecular test performed? ○ Yes ○ No

Date test performed ______________________________

Motive: ______________________________

Dengue ○ Detected ○ Not detected ○ Inconclusive

Ct: ______________________________

Zika ○ Detected ○ Not detected ○ Inconclusive

Ct: ______________________________

Chikungunya ○ Detected ○ Not detected ○ Inconclusive

Ct: ______________________________

SYNOVIAL FLUID - Non-mandatory sampling

Was a synovial fluid sample taken? ○ Yes ○ No

Date taken ______________________________

Time taken ______________________________
(HH:MM)

Motive: ______________________________

Molecular Test: ZDC Zika/Dengue/Chikungunya - SYNOVIAL FLUID	
Molecular test done?	○ Yes ○ No
Date of test	__________________________
Motive:	__________________________
Dengue	○ Detected ○ Not detected ○ Inconclusive
Ct:	__________________________
Zika	○ Detected ○ Not detected ○ Inconclusive
Ct:	__________________________
Chikungunya	○ Detected ○ Not detected ○ Inconclusive
Ct:	_________________________________

Additional comments:	
Notes/ Comments	______________________________________

ADVERSE REACTIONS TO MEDICATIONS

Description of what occurred: ______________________________

Day when occurred:
- ○ 1
- ○ 2
- ○ 3
- ○ 4
- ○ 5
- ○ 6
- ○ 7
- ○ 8
- ○ 9
- ○ 10
- ○ 11
- ○ 12
- ○ 13
- ○ 14
- ○ 15
- ○ 16
- ○ 17
- ○ 18
- ○ 19
- ○ 20
- ○ 21
- ○ 22
- ○ 23
- ○ 24
- ○ 25
- ○ 26
- ○ 27
- ○ 28
- ○ 29
- ○ 30
- ○ 31
- ○ Not known

Month when occurred:
- ○ January
- ○ February
- ○ March
- ○ April
- ○ May
- ○ June
- ○ July
- ○ August
- ○ September
- ○ October
- ○ November
- ○ December
- ○ Not known

Year when occurred: ______________________________

When did it end?

Day ended:

- ◯ 1
- ◯ 2
- ◯ 3
- ◯ 4
- ◯ 5
- ◯ 6
- ◯ 7
- ◯ 8
- ◯ 9
- ◯ 10
- ◯ 11
- ◯ 12
- ◯ 13
- ◯ 14
- ◯ 15
- ◯ 16
- ◯ 17
- ◯ 18
- ◯ 19
- ◯ 20
- ◯ 21
- ◯ 22
- ◯ 23
- ◯ 24
- ◯ 25
- ◯ 26
- ◯ 27
- ◯ 28
- ◯ 29
- ◯ 30
- ◯ 31
- ◯ Not known

Month ended:

- ◯ January
- ◯ February
- ◯ March
- ◯ April
- ◯ May
- ◯ June
- ◯ July
- ◯ August
- ◯ September
- ◯ October
- ◯ November
- ◯ December
- ◯ Not known

Year ended: ______________________________

Did the symptoms last for less than 24 hours? ◯ Yes ◯ No
(Complete only if the symptoms lasted for less than 24 hours)

What time did it begin? ______________________________

What time did it end? ______________________________

Total time in minutes:	______
Amount of suspected medications:	1 2 3
Suspected medication(s):	○ ○ ○ ______
Suspected medication(s):	______
Suspected medication(s):	______
What action was taken in response to the adverse event?	○ Amount has not changed<br>○ Amount increased<br>○ Amount decreased<br>○ Medication temporarily discontinued<br>○ Medication suspended<br>○ Additional treatment (use of other medications, hospitalization)<br>○ Other<br>○ Not reported
Which?	______
Has the medication been reintroduced?	○ Yes ○ No ○ Not known
Did the participant have a similar reaction with the same or similar medication?	○ Yes ○ No ○ Not known
Did the participant require hospitalization or prolonged hospitalization as a result of the event?	○ Yes ○ No ○ Not reported
Did he event cause significant or permanent disability or dysfunction?	○ Yes ○ No ○ Not reported
What significant permanent disability or dysfunction?	______
What was the outcome of the adverse reaction?	○ Fatal ○ No recovery / Not solved<br>○ Recovered / Resolved with side effects<br>○ In recovery ○ Recovered / Solved ○ Not known

SYMPTOMS

Type of symptom:	○ Fever Headache Fatigue<br>○ Pallor skin / mucous membranes<br>○ Jaundice Conjunctival hyperemia<br>○ Visual turbidity Vomiting<br>○ Diarrhea Abdominal pain<br>○ Paraesthesia Dysesthesia<br>○ Arthralgia Edema<br>○ Myalgia Backache<br>○ Itching Cutaneous Rash<br>○ Skin lesions Depression<br>○ Insomnia Changes to memory<br>○ Change in libido Impotency<br>○ Other
Which:	______________________________
Date began:	______________________________
Has this continued until the end of the follow up?	○ Yes ○ No
Date ended:	______________________________

REPLICK - PRE-SCREENING

Pre-screening

Does the participant meet all eligibility criteria?	○ Yes ○ No
Was the consent process performed?	○ Yes ○ No
Justify:	__________________________________
Date consent obtained:	__________________________________

Inclusion criteria

	Yes	No
Is the participant 18 or over?	○	○
Does the participant have a fever (body temperature over or equal to 37.8 ° C) And arthralgia of recent onset (less than or equal to 10 days)	○	○
Does the participant have arthralgia after fever (equal to or less than 7 days), and is suspected of having an arbovirus (Dengue, Chikungunya, Zika)?	○	○

Exclusion Criteria

	Yes	No
Is Participant unavailable for follow-up visits at the research center?	○	○
Does participant evidently have a fever with supposed nonviral origin (cellulitis and abscess)?	○	○
Participant with severe mental disorder that makes it impossible to understand and complete the protocol or does not allow information gathering due to communication disorders, lack of fluency in Portuguese or another language understood by the doctor / assistant / nurse	○	○

Was participant excluded for another reason not specified above?

○ Yes ○ No ○ Não se aplica

Which:

____

Participant PID:

____
